# Crystal structure of 9-(3-bromo-5-chloro-2-hydroxy­phen­yl)-10-(2-hy­droxy­eth­yl)-3,3,6,6-tetra­methyl-3,4,6,7,9,10-hexa­hydro­acridine-1,8(2*H*,5*H*)-dione

**DOI:** 10.1107/S1600536814009556

**Published:** 2014-07-19

**Authors:** Antar A. Abdelhamid, Shaaban K. Mohamed, Jim Simpson

**Affiliations:** a Chemistry Department, Faculty of Science, Sohag University, 82524 Sohag, Egypt; b Chemistry and Environmental Division, Manchester Metropolitan University, Manchester M1 5GD, England; c Department of Chemistry, University of Otago, PO Box 56, Dunedin, New Zealand

**Keywords:** crystal structure, acridine, hydro­acridine

## Abstract

The title compound C_25_H_29_BrClNO_4_, comprises a 3,3,6,6-tetra­methyl­tetra­hydro­acridine-1,8-dione ring system that carries a hy­droxy­ethyl substituent on the acridine N atom and a 3-bromo-5-chloro-2-hy­droxy­phenyl ring on the central methine C atom of the di­hydro­pyridine ring. The benzene ring is inclined to the acridine ring system at an angle of 89.84 (6)° and this conformation is stabilized by an intra­molecular O—H⋯O hydrogen bond between the hy­droxy substituent on the benzene ring and one of the carbonyl groups of the acridinedione unit. In the crystal, O—H⋯O, C—H⋯O and C—H⋯Br hydrogen bonds combine to stack mol­ecules in inter­connected columns propagating along the *a*-axis direction.

## Abstract 2

The structure of a hydroacridine with significant pharmaceutical potential is reported. The acridinone ring system is in the shape of a shallow V with the majority of the ring system substituents on its convex surface; a plethora of classical and non-classical hydrogen bonds stack the molecules into interconnected columns.

## Chemical context   

Acridine derivatives occupy an important position in medicinal chemistry due to their wide range of biological applications. They exhibit fungicidal (Misra & Bahel, 1984 ▶; Srivastava *et al.*, 1985 ▶), anti-cancer (Sondhi *et al.*, 2004 ▶; Sugaya *et al.*, 1994 ▶; Kimura *et al.*, 1993 ▶), anti-parasitic (Ngadi *et al.*, 1993 ▶), anti-inflammatory and anti-microbial (Shul’ga *et al.*, 1974 ▶; Gaiukevich *et al.*, 1973 ▶) activity. They are also components of effective analgesics (Taraporewala & Kauffman, 1990 ▶; Gaidukevich *et al.*, 1987 ▶). Other pharmaceutically active acridine derivatives (*e.g.* Mepacrine, Aza­crine, Proflavine, and Aminacrine) also demonstrate anti­malarial and anti­bacterial activity (Denny *et al.*, 1983 ▶). 
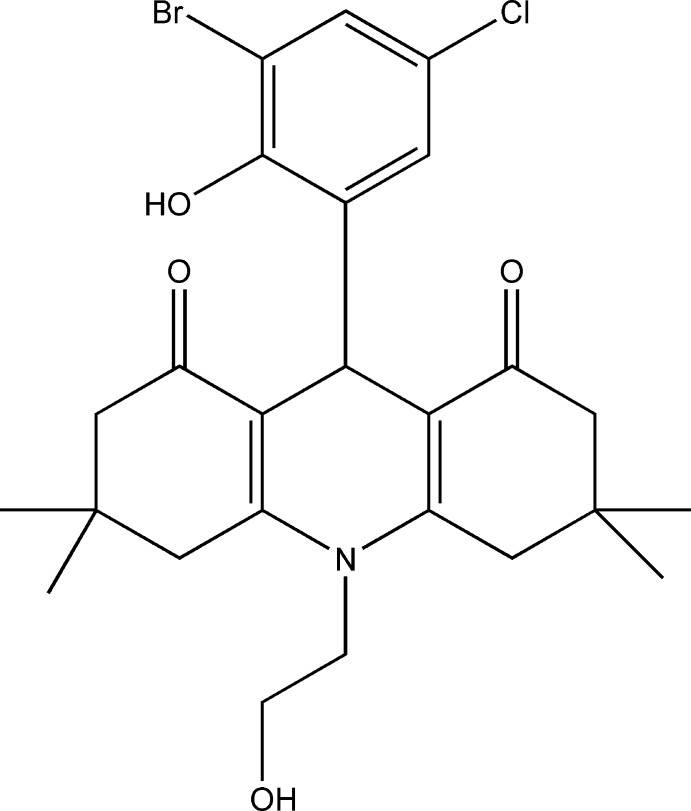
Recently hydro­acridine derivatives were found to have significant anti­microbial activity and to act as potassium channel blockers (Shaikh *et al.*, 2010 ▶; Miyase *et al.*, 2009 ▶). A recent investigation has also shown hydro­acridines to act as inhibitors of sirtuins (class III NAD-dependent de­acetyl­ases) that are considered to be important targets for cancer thera­peutics (Nakhi *et al.*, 2013 ▶). In light of this inter­est and as part of our on-going studies of the synthesis and biological assessment of new hydro­acridinone deriv­atives, we report here the synthesis and crystal structure of the title compound, (1). 

## Structural commentary   

**Figure 1:**
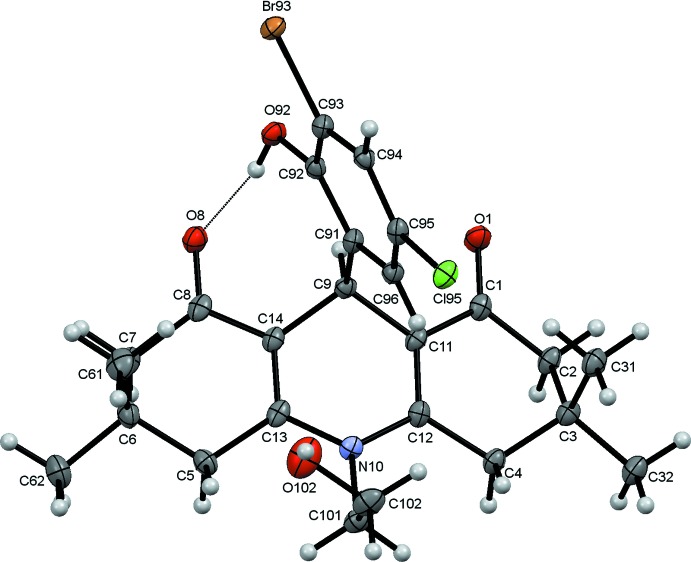
The structure of (1) with ellipsoids drawn at the 50% probability level.

The structure of (1) is shown in Fig. 1 ▶. The 3,3,6,6-tetra­methyl-tetra­hydro­acridine-1,8-dione ring system is substituted at the central methine C9 atom by a 3-bromo-5-chloro-2-hy­droxy­phenyl ring and carries a hy­droxy­ethyl substituent on the acridine N atom. The acridinedione ring system deviates significantly from planarity with an r.m.s. deviation of 0.336 Å for the 13 C atoms and one N atom of the acridine unit. This plane is almost orthogonal to the benzene ring plane [dihedral angle = 89.84 (6)°], a conformation that is stabilized by a strong intra­molecular O92—H92⋯O8 hydrogen bond between the two systems (Table 1 ▶). Both the 3-bromo-5-chloro-2-hy­droxy­phenyl and hy­droxy­ethyl substituents point in the same direction with respect to the acridine plane. Furthermore, one methyl group is axial and the other equatorial with respect to the two outer cyclo­hexenone rings of the acridinedione and again, the axial methyl substituents are found on the same face of the acridinedione ring system. Overall this ring system is V-shaped with the substituents mentioned above on the convex surface of the shallow V. The outer cyclo­hexenone rings both adopt flattened chair configurations with the C3 and C6 atoms each 0.646 (4) Å, in the same direction, from the best-fit planes through the remaining five C atoms. In contrast, the central C9/N10/C11–C14 ring can best be described as a flattened boat with C9 and N10 0.423 (4) and 0.154 (4) Å, respectively, from the best-fit plane through the remaining four C atoms. The bond lengths and angles in the mol­ecule of (1) agree reasonably well with those found in closely related mol­ecules (Abdelhamid *et al.*, 2011 ▶; Khalilov *et al.*, 2011 ▶).

## Supra­molecular features   

**Figure 2:**
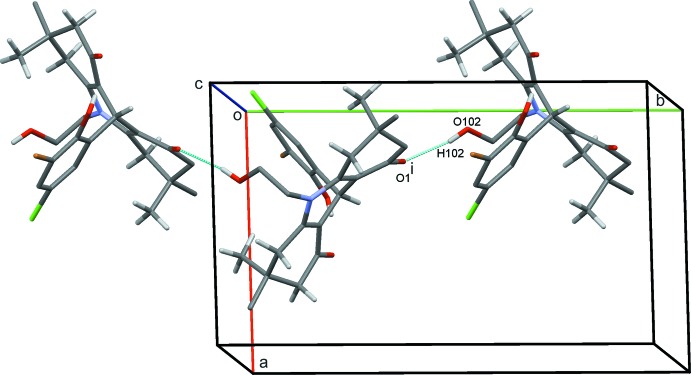
Zigzag chains of (1) parallel to the *b* axis with hydrogen bonds drawn as dashed lines and symmetry operations shown in Table 1 ▶. For clarity, H atoms bound to atoms not involved in hydrogen bonding are not shown.

**Figure 3:**
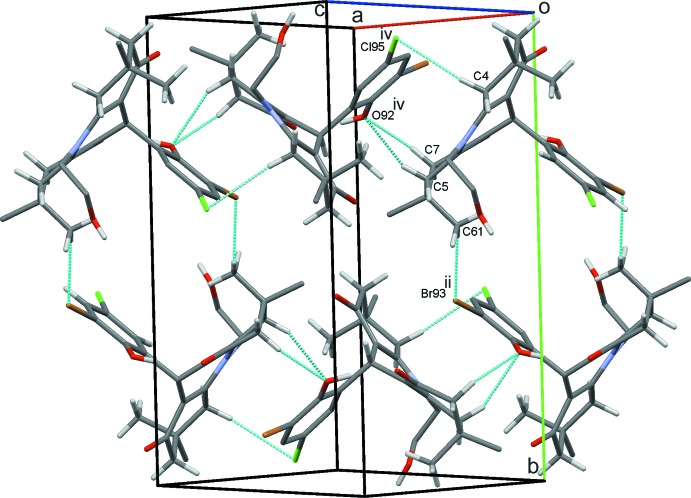
Sheets of mol­ecules of (1) parallel to (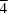
21) with hydrogen bonds drawn as dashed lines and symmetry operations shown in Table 1 ▶. For clarity, H atoms bound to atoms not involved in hydrogen bonding are not shown.

**Figure 4:**
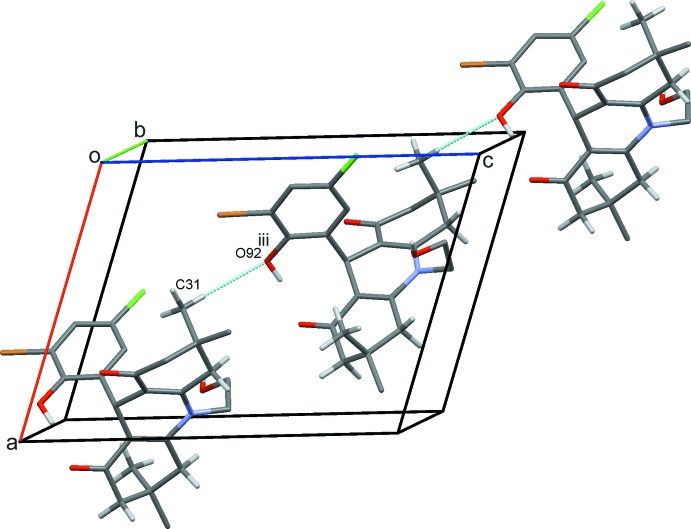
Chains of mol­ecules of (1) along the diagonal of the *ac* plane with hydrogen bonds drawn as dashed lines and symmetry operations shown in Table 1 ▶. For clarity, H atoms bound to atoms not involved in hydrogen bonding are not shown.

**Figure 5:**
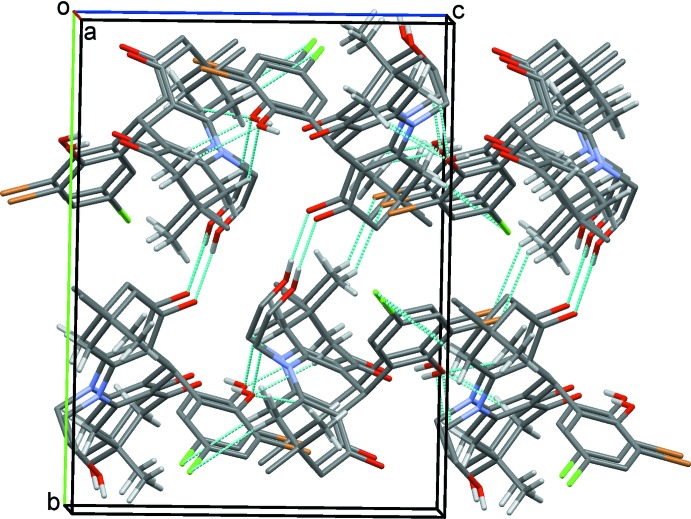
Overall packing for (1) viewed along the *a* axis with hydrogen bonds drawn as dashed lines.

The crystal structure of (1) features O102—H102⋯O1 hydrogen bonds, which link the mol­ecules into zigzag chains parallel to the *b* axis (Fig. 2 ▶). Weak C4—H4*A*⋯Cl95 together with C5—H5*B*⋯O92 and C7—H7*A*⋯O92 hydrogen bonds to the same acceptor oxygen atom form 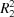
(15), 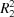
(13) and 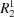
(6) rings. These, combined with weaker inversion-related C61—H61*B*⋯Br93 contacts [which in turn generate 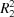
(22) motifs], generate sheets of mol­ecules lying parallel to the (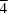
21) plane, as shown in Fig. 3 ▶. C31—H31*B*⋯O92 hydrogen bonds form additional chains of mol­ecules along the *ac* diagonal (Fig. 4 ▶). Overall, these inter­actions stack the mol­ecules into inter­connected columns along the *a*-axis direction (Fig. 5 ▶).

## Database survey   

Numerous structures of acridine and its derivatives have been reported previously, with 373 entries in the current database (Version 5.35, November 2013 with 1 update; Allen, 2002 ▶). However, far fewer structures of derivatives of the seminal hydro­acridine, 3,3,6,6-tetra­methyl-3,4,6,7,9,10-hexa­hydro-1,8(2*H*,5*H*)-acridinedione (Natarajan & Mathews, 2011 ▶) are found with only 25 unique structures of derivatives with an aryl substituent on the methine C atom and an alkyl or aryl substituent on the N atom. Of these, aromatic substituents on the N atom predominate with 15 entries (see, for example, Nakhi *et al.* 2013 ▶; Shi *et al.* 2008 ▶; Wang *et al.* 2003 ▶). Two structures, 10-(2-hy­droxy­eth­yl)-9-(2-hy­droxy­phen­yl)-3,3,6,6-tetra­methyl-1,2,3,4,5,6,7,8,9,10-deca­hydro­acridine-1,8-dione (Abdelhamid *et al.*, 2011 ▶) and 9-(5-bromo-2-hy­droxy­phen­yl)-10-(2-hy­droxy­prop­yl)-3,3,6,6-tetra­methyl-1,2,3,4,5,6,7,8,9,10-deca­hydro­acridine-1,8-dione (Khalilov *et al.*, 2011 ▶) closely resemble (1), each with 2-hy­droxy substituents on the aromatic rings that form intra­molecular hydrogen bonds to one of the two keto O atoms in each mol­ecule. In the first instance, the 2-hy­droxy­ethyl substituent on the N atom is identical to that for (1), while the 2-hy­droxy­propyl substituent in the second analogue is closely related.

## Synthesis and crystallization   

A mixture of 1 mmol (235.5 mg) 3-bromo-5-chloro-2-hy­droxy­benzaldehyde, 2 mmol (280 mg) 5,5-di­methyl­cyclo­hexane-1,3-dione and 1 mmol (61 mg) amino-ethanol in 30 ml of ethanol was refluxed for 12 h. The reaction was monitored by TLC until completion. Excess solvent was evaporated under vacuum and the resulting solid product was recrystallized from a mixture of ethanol/acetone (10:1 *v*:*v*) to afford yellow needles of the title compound. M.p. 513 K, 82% yield.

IR cm^−1^: OH phenolic 3400, OH alcoholic 3335, Ar 3001, CH-aliphatic 2882, CO 1694, C=C 1591, C—Br 605, C—Cl 738; ^1^H NMR: δ 10.01 (*s*, 1H, OH phenolic), 7.3 (*d*, 2H, Ar), 6.7 (*d*, 1H, C9), 5.00 (*s*, 1H, OH alcoholic), 4.02 (*t*, 2H, C2), 3.75 (*t*, 2H, C7), 2.95 (*d*, 2H, C4), 2.7(*d*, 2H, C5), 2.2 (*m*, 4H, ethyl group), 1–1.2 (*m*, 12H, 4 methyl groups); ^13^C NMR: δ 199, 200 (C=O, C1, C8), 145, 132 and 130 (C=C Ar), 110, 112 (C=C, in acridine fused rings), 122 (C—N), 62 (C—Br), 73 (C—Cl), 50 (C—OH), 20, 28, 30 and 32 (C—C of CH_2_CH_2_ and 4CH_3_); MS: *m*/*z* 522 (100), 523 (30), 524 (100), 525 (30), 443 (56), 363 (39), 271 (42), 175 (29), 94 (74). Analysis calculated for C_25_H_29_BrClNO_4_ (522.85): C 57.43, H 5.59, Br 15.28, Cl 6.78, N 2.68%; found: C 57.41, H 5.60, Br 15.31, Cl 6.81, N 2.71.

## Refinement   

Crystal data, data collection and structure refinement details are summarized in Table 2 ▶. The H atoms of the two hy­droxy substituents were located in an electron density map and their coordinates were freely refined with *U*_iso_ = 1.5*U*_eq_ (O). All H atoms bound to carbon were refined using a riding model with *d*(C—H) = 0.95 Å *U*_iso_ = 1.2*U*_eq_ (C) for aromatic, 0.99 Å, *U*_iso_ = 1.2*U*_eq_ (C) for methyl­ene, 1.00 Å, *U*_iso_ = 1.2*U*_eq_ (C) for methine, and 0.98 Å, *U*_iso_ = 1.5*U*_eq_ (C) for methyl H atoms.

## Supplementary Material

Crystal structure: contains datablock(s) global, 1. DOI: 10.1107/S1600536814009556/hb0002sup1.cif

Structure factors: contains datablock(s) I. DOI: 10.1107/S1600536814009556/hb0002Isup2.hkl

CCDC reference: 1004259

Additional supporting information:  crystallographic information; 3D view; checkCIF report

## Figures and Tables

**Table 2:** Experimental details

Crystal data	
Chemical formula	C_25_H_29_BrClNO_4_
*M*_r_	522.85
Crystal system, space group	Monoclinic, *P*2_1_/*n*
Temperature (K)	100
*a*, *b*, *c* (Å)	10.5373 (3), 17.1597 (3), 13.7278 (4)
β (°)	107.908 (3)
*V* (Å^3^)	2361.96 (10)
*Z*	4
Radiation type	Cu *K*α
μ (mm^−1^)	3.67
Crystal size (mm)	0.19 × 0.07 × 0.06

Data collection	
Diffractometer	Agilent SuperNova (Dual, Cu at zero, Atlas)
Absorption correction	Multi-scan (*CrysAlis PRO*; Agilent, 2013 ▶)
*T*_min_, *T*_max_	0.733, 1.000
No. of measured, independent and observed [*I* > 2σ(*I*)] reflections	20233, 4922, 4128
*R*_int_	0.076
(sin θ/λ)_max_ (Å^−1^)	0.631

Refinement	
*R*[*F* ^2^ > 2σ(*F* ^2^)], *wR*(*F* ^2^), *S*	0.040, 0.108, 1.03
No. of reflections	4922
No. of parameters	299
H-atom treatment	H atoms treated by a mixture of independent and constrained refinement
Δρ_max_, Δρ_min_ (e Å^−3^)	0.67, −0.57

Computer programs: *CrysAlis PRO* (Agilent, 2013 ▶), *SHELXS97* and *SHELXL2013* (Sheldrick, 2008 ▶), *TITAN2000* (Hunter & Simpson, 1999 ▶), *Mercury* (Macrae *et al.*, 2008 ▶), *enCIFer* (Allen *et al.*, 2004 ▶), *PLATON* (Spek, 2009 ▶) and *publCIF* (Westrip, 2010 ▶).

**Table 1:** Hydrogen-bond geometry (Å, °)

*D*—H⋯*A*	*D*—H	H⋯*A*	*D*⋯*A*	*D*—H⋯*A*
O92—H92⋯O8	0.82 (4)	1.81 (4)	2.613 (3)	166 (4)
O102—H102⋯O1^i^	0.81 (5)	2.01 (5)	2.808 (3)	167 (5)
C61—H61*B*⋯Br93^ii^	0.98	2.87	3.720 (3)	146
C31—H31*B*⋯O92^iii^	0.98	2.65	3.532 (4)	150
C5—H5*B*⋯O92^iv^	0.99	2.71	3.479 (4)	135
C7—H7*A*⋯O92^iv^	0.99	2.44	3.346 (4)	151
C4—H4*A*⋯Cl95^iv^	0.99	2.88	3.868 (3)	173

Symmetry codes: (i) 
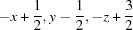
; (ii) 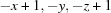; (iii) 
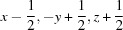
; (iv) 
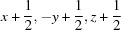
.

## supplementary crystallographic information

### Crystal data

**Table:**

C_25_H_29_BrClNO_4_	*F*(000) = 1080
*M**_r_* = 522.85	*D*_x_ = 1.470 Mg m^−^^3^
Monoclinic, *P*2_1_/*n*	Cu *K*α radiation, λ = 1.54184 Å
*a* = 10.5373 (3) Å	Cell parameters from 8397 reflections
*b* = 17.1597 (3) Å	θ = 4.3–76.4°
*c* = 13.7278 (4) Å	µ = 3.67 mm^−^^1^
β = 107.908 (3)°	*T* = 100 K
*V* = 2361.96 (10) Å^3^	Needle, yellow
*Z* = 4	0.19 × 0.07 × 0.06 mm

### Data collection

**Table:**

Agilent SuperNova (Dual, Cu at zero, Atlas) diffractometer	4922 independent reflections
Radiation source: SuperNova (Cu) X-ray Source	4128 reflections with *I* > 2σ(*I*)
Detector resolution: 5.1725 pixels mm^-1^	*R*_int_ = 0.076
ω scans	θ_max_ = 76.7°, θ_min_ = 4.3°
Absorption correction: multi-scan (*CrysAlis PRO*; Agilent, 2013)	*h* = −13→12
*T*_min_ = 0.733, *T*_max_ = 1.000	*k* = −21→21
20233 measured reflections	*l* = −17→15

### Refinement

**Table:**

Refinement on *F*^2^	0 restraints
Least-squares matrix: full	Hydrogen site location: mixed
*R*[*F*^2^ > 2σ(*F*^2^)] = 0.040	H atoms treated by a mixture of independent and constrained refinement
*wR*(*F*^2^) = 0.108	*w* = 1/[σ^2^(*F*_o_^2^) + (0.0431*P*)^2^ + 3.0935*P*] where *P* = (*F*_o_^2^ + 2*F*_c_^2^)/3
*S* = 1.03	(Δ/σ)_max_ = 0.001
4922 reflections	Δρ_max_ = 0.67 e Å^−^^3^
299 parameters	Δρ_min_ = −0.57 e Å^−^^3^

### Special details

**Table:**

Geometry. All e.s.d.'s (except the e.s.d. in the dihedral angle between two l.s. planes) are estimated using the full covariance matrix. The cell e.s.d.'s are taken into account individually in the estimation of e.s.d.'s in distances, angles and torsion angles; correlations between e.s.d.'s in cell parameters are only used when they are defined by crystal symmetry. An approximate (isotropic) treatment of cell e.s.d.'s is used for estimating e.s.d.'s involving l.s. planes.

### Fractional atomic coordinates and isotropic or equivalent isotropic displacement parameters (Å^2^)

**Table:**

	*x*	*y*	*z*	*U*_iso_*/*U*_eq_	
C1	0.2756 (3)	0.38420 (16)	0.7425 (2)	0.0165 (5)	
O1	0.2646 (2)	0.41046 (12)	0.65603 (15)	0.0220 (4)	
C2	0.2254 (3)	0.42807 (16)	0.8181 (2)	0.0183 (6)	
H2A	0.1555	0.4653	0.7808	0.022*	
H2B	0.2996	0.4584	0.8644	0.022*	
C3	0.1680 (3)	0.37339 (17)	0.8817 (2)	0.0182 (6)	
C31	0.0446 (3)	0.33120 (18)	0.8117 (2)	0.0218 (6)	
H31A	−0.0221	0.3697	0.7760	0.033*	
H31B	0.0068	0.2973	0.8531	0.033*	
H31C	0.0707	0.2997	0.7615	0.033*	
C32	0.1278 (3)	0.42057 (18)	0.9624 (2)	0.0224 (6)	
H32A	0.2058	0.4486	1.0058	0.034*	
H32B	0.0938	0.3852	1.0046	0.034*	
H32C	0.0582	0.4580	0.9282	0.034*	
C4	0.2757 (3)	0.31371 (16)	0.9365 (2)	0.0169 (5)	
H4A	0.3401	0.3394	0.9958	0.020*	
H4B	0.2327	0.2709	0.9631	0.020*	
C5	0.6375 (3)	0.13867 (17)	0.9129 (2)	0.0187 (6)	
H5A	0.5984	0.0944	0.9400	0.022*	
H5B	0.6987	0.1665	0.9719	0.022*	
C6	0.7183 (3)	0.10665 (17)	0.8456 (2)	0.0199 (6)	
C61	0.6375 (3)	0.04386 (18)	0.7733 (2)	0.0272 (7)	
H61A	0.6193	0.0006	0.8137	0.041*	
H61B	0.6886	0.0247	0.7294	0.041*	
H61C	0.5531	0.0661	0.7307	0.041*	
C62	0.8489 (3)	0.0709 (2)	0.9150 (3)	0.0284 (7)	
H62A	0.8281	0.0300	0.9576	0.043*	
H62B	0.9024	0.1115	0.9591	0.043*	
H62C	0.8993	0.0483	0.8726	0.043*	
C7	0.7509 (3)	0.17336 (18)	0.7830 (2)	0.0210 (6)	
H7A	0.8091	0.2117	0.8300	0.025*	
H7B	0.8000	0.1525	0.7377	0.025*	
C8	0.6261 (3)	0.21319 (16)	0.7195 (2)	0.0179 (5)	
O8	0.6181 (2)	0.23890 (12)	0.63285 (15)	0.0211 (4)	
C9	0.3948 (3)	0.26576 (16)	0.7015 (2)	0.0146 (5)	
H9	0.4202	0.3042	0.6560	0.018*	
N10	0.4338 (2)	0.21496 (13)	0.90561 (17)	0.0150 (4)	
C101	0.4242 (3)	0.17121 (17)	0.9961 (2)	0.0193 (6)	
H10A	0.3974	0.2072	1.0427	0.023*	
H10B	0.5128	0.1494	1.0337	0.023*	
C102	0.3240 (3)	0.10579 (18)	0.9650 (2)	0.0243 (6)	
H10C	0.3186	0.0776	1.0265	0.029*	
H10D	0.2347	0.1272	0.9288	0.029*	
O102	0.3641 (3)	0.05431 (14)	0.9002 (2)	0.0338 (6)	
H102	0.318 (5)	0.015 (3)	0.888 (4)	0.051*	
C11	0.3399 (3)	0.30933 (16)	0.7750 (2)	0.0151 (5)	
C12	0.3510 (3)	0.27926 (16)	0.8694 (2)	0.0156 (5)	
C13	0.5263 (3)	0.19368 (15)	0.8568 (2)	0.0153 (5)	
C14	0.5183 (3)	0.22261 (16)	0.7631 (2)	0.0155 (5)	
C91	0.2933 (3)	0.20862 (16)	0.6350 (2)	0.0152 (5)	
C92	0.2973 (3)	0.18932 (16)	0.5361 (2)	0.0155 (5)	
O92	0.3860 (2)	0.22101 (12)	0.49447 (15)	0.0185 (4)	
H92	0.453 (4)	0.232 (2)	0.542 (3)	0.028*	
C93	0.2039 (3)	0.13599 (17)	0.4773 (2)	0.0167 (5)	
Br93	0.20688 (3)	0.10910 (2)	0.34475 (2)	0.01878 (10)	
C94	0.1101 (3)	0.10051 (15)	0.5147 (2)	0.0156 (5)	
H94	0.0464	0.0652	0.4736	0.019*	
C95	0.1116 (3)	0.11794 (16)	0.6140 (2)	0.0165 (5)	
Cl95	0.00165 (7)	0.07019 (4)	0.66614 (5)	0.01978 (15)	
C96	0.2010 (3)	0.17139 (16)	0.6729 (2)	0.0157 (5)	
H96	0.1993	0.1828	0.7402	0.019*	

### Atomic displacement parameters (Å^2^)

**Table:**

	*U*^11^	*U*^22^	*U*^33^	*U*^12^	*U*^13^	*U*^23^
C1	0.0146 (12)	0.0157 (13)	0.0207 (13)	−0.0030 (10)	0.0075 (10)	−0.0029 (10)
O1	0.0287 (11)	0.0196 (10)	0.0212 (10)	0.0037 (9)	0.0128 (8)	0.0039 (8)
C2	0.0217 (14)	0.0140 (13)	0.0223 (13)	−0.0013 (11)	0.0114 (11)	−0.0024 (11)
C3	0.0179 (13)	0.0196 (13)	0.0190 (13)	−0.0008 (11)	0.0085 (11)	−0.0019 (11)
C31	0.0183 (14)	0.0254 (15)	0.0241 (14)	−0.0015 (12)	0.0100 (11)	−0.0023 (12)
C32	0.0257 (15)	0.0213 (14)	0.0243 (14)	0.0003 (12)	0.0139 (12)	−0.0013 (12)
C4	0.0182 (13)	0.0160 (13)	0.0190 (12)	−0.0009 (11)	0.0094 (10)	−0.0004 (11)
C5	0.0184 (14)	0.0154 (13)	0.0208 (13)	0.0016 (11)	0.0039 (10)	0.0017 (11)
C6	0.0187 (14)	0.0177 (14)	0.0230 (14)	0.0046 (11)	0.0058 (11)	−0.0007 (11)
C61	0.0315 (17)	0.0190 (14)	0.0308 (16)	0.0009 (13)	0.0094 (13)	−0.0066 (13)
C62	0.0243 (16)	0.0259 (16)	0.0339 (17)	0.0087 (13)	0.0070 (13)	0.0032 (13)
C7	0.0176 (14)	0.0244 (15)	0.0235 (14)	0.0034 (12)	0.0099 (11)	−0.0004 (12)
C8	0.0187 (13)	0.0147 (13)	0.0213 (13)	−0.0036 (11)	0.0076 (10)	−0.0067 (11)
O8	0.0183 (10)	0.0254 (11)	0.0215 (10)	−0.0003 (8)	0.0089 (8)	−0.0010 (8)
C9	0.0163 (13)	0.0143 (12)	0.0145 (12)	0.0000 (10)	0.0066 (10)	0.0010 (10)
N10	0.0173 (11)	0.0119 (10)	0.0176 (11)	−0.0013 (9)	0.0082 (9)	0.0013 (9)
C101	0.0253 (14)	0.0169 (13)	0.0178 (13)	−0.0004 (11)	0.0100 (11)	0.0008 (11)
C102	0.0328 (17)	0.0195 (14)	0.0246 (15)	−0.0041 (13)	0.0145 (13)	0.0021 (11)
O102	0.0382 (14)	0.0207 (11)	0.0491 (15)	−0.0109 (10)	0.0232 (11)	−0.0082 (11)
C11	0.0168 (13)	0.0129 (12)	0.0170 (12)	−0.0023 (10)	0.0074 (10)	−0.0034 (10)
C12	0.0145 (13)	0.0132 (12)	0.0205 (13)	−0.0020 (10)	0.0077 (10)	−0.0016 (10)
C13	0.0156 (12)	0.0122 (12)	0.0204 (12)	−0.0055 (10)	0.0087 (10)	−0.0060 (10)
C14	0.0149 (13)	0.0141 (12)	0.0186 (12)	−0.0021 (10)	0.0067 (10)	−0.0037 (10)
C91	0.0148 (12)	0.0143 (12)	0.0176 (12)	0.0014 (10)	0.0067 (10)	−0.0009 (10)
C92	0.0160 (13)	0.0138 (12)	0.0175 (12)	0.0036 (10)	0.0061 (10)	0.0024 (10)
O92	0.0172 (10)	0.0237 (10)	0.0167 (9)	−0.0020 (8)	0.0084 (8)	−0.0001 (8)
C93	0.0160 (13)	0.0177 (13)	0.0169 (12)	0.0026 (11)	0.0058 (10)	−0.0007 (10)
Br93	0.02276 (17)	0.01832 (16)	0.01709 (15)	0.00042 (11)	0.00884 (11)	−0.00280 (10)
C94	0.0146 (13)	0.0133 (12)	0.0177 (12)	0.0010 (10)	0.0034 (10)	−0.0010 (10)
C95	0.0160 (13)	0.0130 (12)	0.0225 (13)	0.0008 (10)	0.0089 (10)	0.0023 (10)
Cl95	0.0189 (3)	0.0198 (3)	0.0234 (3)	−0.0044 (3)	0.0106 (2)	−0.0021 (3)
C96	0.0167 (13)	0.0150 (12)	0.0163 (12)	0.0017 (10)	0.0066 (10)	0.0001 (10)

### Geometric parameters (Å, º)

**Table:**

C1—O1	1.242 (3)	C7—H7A	0.9900
C1—C11	1.457 (4)	C7—H7B	0.9900
C1—C2	1.504 (4)	C8—O8	1.247 (4)
C2—C3	1.527 (4)	C8—C14	1.447 (4)
C2—H2A	0.9900	C9—C11	1.506 (4)
C2—H2B	0.9900	C9—C14	1.510 (4)
C3—C32	1.532 (4)	C9—C91	1.529 (4)
C3—C31	1.539 (4)	C9—H9	1.0000
C3—C4	1.542 (4)	N10—C13	1.392 (3)
C31—H31A	0.9800	N10—C12	1.399 (4)
C31—H31B	0.9800	N10—C101	1.481 (3)
C31—H31C	0.9800	C101—C102	1.510 (4)
C32—H32A	0.9800	C101—H10A	0.9900
C32—H32B	0.9800	C101—H10B	0.9900
C32—H32C	0.9800	C102—O102	1.408 (4)
C4—C12	1.508 (4)	C102—H10C	0.9900
C4—H4A	0.9900	C102—H10D	0.9900
C4—H4B	0.9900	O102—H102	0.81 (5)
C5—C13	1.517 (4)	C11—C12	1.367 (4)
C5—C6	1.538 (4)	C13—C14	1.357 (4)
C5—H5A	0.9900	C91—C96	1.392 (4)
C5—H5B	0.9900	C91—C92	1.410 (4)
C6—C7	1.532 (4)	C92—O92	1.351 (3)
C6—C61	1.533 (4)	C92—C93	1.403 (4)
C6—C62	1.541 (4)	O92—H92	0.82 (4)
C61—H61A	0.9800	C93—C94	1.387 (4)
C61—H61B	0.9800	C93—Br93	1.887 (3)
C61—H61C	0.9800	C94—C95	1.390 (4)
C62—H62A	0.9800	C94—H94	0.9500
C62—H62B	0.9800	C95—C96	1.384 (4)
C62—H62C	0.9800	C95—Cl95	1.742 (3)
C7—C8	1.501 (4)	C96—H96	0.9500
			
O1—C1—C11	120.7 (3)	C8—C7—H7B	109.4
O1—C1—C2	121.9 (3)	C6—C7—H7B	109.4
C11—C1—C2	117.4 (2)	H7A—C7—H7B	108.0
C1—C2—C3	111.8 (2)	O8—C8—C14	121.5 (3)
C1—C2—H2A	109.2	O8—C8—C7	120.4 (3)
C3—C2—H2A	109.2	C14—C8—C7	118.0 (2)
C1—C2—H2B	109.2	C11—C9—C14	108.1 (2)
C3—C2—H2B	109.2	C11—C9—C91	112.1 (2)
H2A—C2—H2B	107.9	C14—C9—C91	110.1 (2)
C2—C3—C32	109.5 (2)	C11—C9—H9	108.8
C2—C3—C31	109.8 (2)	C14—C9—H9	108.8
C32—C3—C31	109.4 (2)	C91—C9—H9	108.8
C2—C3—C4	109.0 (2)	C13—N10—C12	119.2 (2)
C32—C3—C4	108.9 (2)	C13—N10—C101	120.6 (2)
C31—C3—C4	110.2 (2)	C12—N10—C101	120.1 (2)
C3—C31—H31A	109.5	N10—C101—C102	111.2 (2)
C3—C31—H31B	109.5	N10—C101—H10A	109.4
H31A—C31—H31B	109.5	C102—C101—H10A	109.4
C3—C31—H31C	109.5	N10—C101—H10B	109.4
H31A—C31—H31C	109.5	C102—C101—H10B	109.4
H31B—C31—H31C	109.5	H10A—C101—H10B	108.0
C3—C32—H32A	109.5	O102—C102—C101	109.0 (3)
C3—C32—H32B	109.5	O102—C102—H10C	109.9
H32A—C32—H32B	109.5	C101—C102—H10C	109.9
C3—C32—H32C	109.5	O102—C102—H10D	109.9
H32A—C32—H32C	109.5	C101—C102—H10D	109.9
H32B—C32—H32C	109.5	H10C—C102—H10D	108.3
C12—C4—C3	114.2 (2)	C102—O102—H102	112 (3)
C12—C4—H4A	108.7	C12—C11—C1	121.3 (2)
C3—C4—H4A	108.7	C12—C11—C9	120.6 (2)
C12—C4—H4B	108.7	C1—C11—C9	118.1 (2)
C3—C4—H4B	108.7	C11—C12—N10	119.8 (2)
H4A—C4—H4B	107.6	C11—C12—C4	121.5 (2)
C13—C5—C6	113.6 (2)	N10—C12—C4	118.7 (2)
C13—C5—H5A	108.8	C14—C13—N10	120.8 (3)
C6—C5—H5A	108.8	C14—C13—C5	121.5 (3)
C13—C5—H5B	108.8	N10—C13—C5	117.7 (2)
C6—C5—H5B	108.8	C13—C14—C8	121.5 (3)
H5A—C5—H5B	107.7	C13—C14—C9	120.1 (2)
C7—C6—C61	109.8 (2)	C8—C14—C9	118.3 (2)
C7—C6—C5	109.3 (2)	C96—C91—C92	118.9 (2)
C61—C6—C5	109.9 (2)	C96—C91—C9	120.8 (2)
C7—C6—C62	109.4 (3)	C92—C91—C9	120.2 (2)
C61—C6—C62	109.4 (2)	O92—C92—C93	118.2 (2)
C5—C6—C62	109.0 (2)	O92—C92—C91	122.8 (2)
C6—C61—H61A	109.5	C93—C92—C91	118.9 (3)
C6—C61—H61B	109.5	C92—O92—H92	107 (3)
H61A—C61—H61B	109.5	C94—C93—C92	121.8 (3)
C6—C61—H61C	109.5	C94—C93—Br93	118.4 (2)
H61A—C61—H61C	109.5	C92—C93—Br93	119.8 (2)
H61B—C61—H61C	109.5	C93—C94—C95	118.3 (2)
C6—C62—H62A	109.5	C93—C94—H94	120.9
C6—C62—H62B	109.5	C95—C94—H94	120.9
H62A—C62—H62B	109.5	C96—C95—C94	121.1 (3)
C6—C62—H62C	109.5	C96—C95—Cl95	119.4 (2)
H62A—C62—H62C	109.5	C94—C95—Cl95	119.5 (2)
H62B—C62—H62C	109.5	C95—C96—C91	120.9 (3)
C8—C7—C6	111.0 (2)	C95—C96—H96	119.5
C8—C7—H7A	109.4	C91—C96—H96	119.5
C6—C7—H7A	109.4		
			
O1—C1—C2—C3	143.8 (3)	C101—N10—C13—C14	166.6 (2)
C11—C1—C2—C3	−36.9 (3)	C12—N10—C13—C5	164.2 (2)
C1—C2—C3—C32	175.9 (2)	C101—N10—C13—C5	−14.7 (4)
C1—C2—C3—C31	−63.9 (3)	C6—C5—C13—C14	−12.2 (4)
C1—C2—C3—C4	56.9 (3)	C6—C5—C13—N10	169.1 (2)
C2—C3—C4—C12	−44.4 (3)	N10—C13—C14—C8	168.2 (2)
C32—C3—C4—C12	−163.8 (2)	C5—C13—C14—C8	−10.4 (4)
C31—C3—C4—C12	76.1 (3)	N10—C13—C14—C9	−11.8 (4)
C13—C5—C6—C7	45.1 (3)	C5—C13—C14—C9	169.5 (2)
C13—C5—C6—C61	−75.5 (3)	O8—C8—C14—C13	179.1 (3)
C13—C5—C6—C62	164.6 (2)	C7—C8—C14—C13	−2.7 (4)
C61—C6—C7—C8	63.6 (3)	O8—C8—C14—C9	−0.9 (4)
C5—C6—C7—C8	−57.0 (3)	C7—C8—C14—C9	177.3 (2)
C62—C6—C7—C8	−176.2 (2)	C11—C9—C14—C13	33.7 (3)
C6—C7—C8—O8	−144.6 (3)	C91—C9—C14—C13	−89.1 (3)
C6—C7—C8—C14	37.2 (3)	C11—C9—C14—C8	−146.4 (2)
C13—N10—C101—C102	−91.2 (3)	C91—C9—C14—C8	90.8 (3)
C12—N10—C101—C102	89.9 (3)	C11—C9—C91—C96	−32.5 (3)
N10—C101—C102—O102	60.0 (3)	C14—C9—C91—C96	87.9 (3)
O1—C1—C11—C12	−179.1 (3)	C11—C9—C91—C92	151.4 (2)
C2—C1—C11—C12	1.6 (4)	C14—C9—C91—C92	−88.2 (3)
O1—C1—C11—C9	1.2 (4)	C96—C91—C92—O92	−178.2 (2)
C2—C1—C11—C9	−178.1 (2)	C9—C91—C92—O92	−1.9 (4)
C14—C9—C11—C12	−33.5 (3)	C96—C91—C92—C93	3.0 (4)
C91—C9—C11—C12	88.1 (3)	C9—C91—C92—C93	179.2 (2)
C14—C9—C11—C1	146.2 (2)	O92—C92—C93—C94	179.6 (2)
C91—C9—C11—C1	−92.2 (3)	C91—C92—C93—C94	−1.5 (4)
C1—C11—C12—N10	−168.3 (2)	O92—C92—C93—Br93	1.1 (3)
C9—C11—C12—N10	11.4 (4)	C91—C92—C93—Br93	180.0 (2)
C1—C11—C12—C4	11.8 (4)	C92—C93—C94—C95	−1.1 (4)
C9—C11—C12—C4	−168.5 (2)	Br93—C93—C94—C95	177.4 (2)
C13—N10—C12—C11	14.7 (4)	C93—C94—C95—C96	2.3 (4)
C101—N10—C12—C11	−166.4 (2)	C93—C94—C95—Cl95	−176.3 (2)
C13—N10—C12—C4	−165.4 (2)	C94—C95—C96—C91	−0.8 (4)
C101—N10—C12—C4	13.5 (4)	Cl95—C95—C96—C91	177.8 (2)
C3—C4—C12—C11	11.1 (4)	C92—C91—C96—C95	−1.9 (4)
C3—C4—C12—N10	−168.8 (2)	C9—C91—C96—C95	−178.1 (2)
C12—N10—C13—C14	−14.5 (4)		

### Hydrogen-bond geometry (Å, º)

**Table:**

*D*—H···*A*	*D*—H	H···*A*	*D*···*A*	*D*—H···*A*
O92—H92···O8	0.82 (4)	1.81 (4)	2.613 (3)	166 (4)
O102—H102···O1^i^	0.81 (5)	2.01 (5)	2.808 (3)	167 (5)
C61—H61*B*···Br93^ii^	0.98	2.87	3.720 (3)	146
C31—H31*B*···O92^iii^	0.98	2.65	3.532 (4)	150
C5—H5*B*···O92^iv^	0.99	2.71	3.479 (4)	135
C7—H7*A*···O92^iv^	0.99	2.44	3.346 (4)	151
C4—H4*A*···Cl95^iv^	0.99	2.88	3.868 (3)	173

Symmetry codes: (i) −*x*+1/2, *y*−1/2, −*z*+3/2; (ii) −*x*+1, −*y*, −*z*+1; (iii) *x*−1/2, −*y*+1/2, *z*+1/2; (iv) *x*+1/2, −*y*+1/2, *z*+1/2.
